# Investigating an objective orthodontics index in order to screen body dysmorphic disorder, a case-control study in orthodontic patients

**DOI:** 10.1192/j.eurpsy.2023.864

**Published:** 2023-07-19

**Authors:** S. Omidvar Tehrani, A. Talaei, F. Farzanegan

**Affiliations:** 1 Psychiatry and Behavioral Sciences Research Center; 2 Dental Materials Research Center, School of Dentistry, Mashhad University of Medical Sciences, Mashhad, Iran, Islamic Republic Of

## Abstract

**Introduction:**

Recently, orthognathic surgeries have gained popularity in orthodontics settings. The perception of body image is a driving force in individuals who seek orthodontic treatments. Therefore, the clinician should be suspicious of underlying psychological conditions, namely body dysmorphic disorder (BDD). Indices like the “index of complexity, outcome, and need” (ICON) in orthodontics not only objectively determine malocclusion traits but also consider the influence of subjective beauty perspectives.

**Objectives:**

This study aimed to assess if dentists can use an objective orthodontics index in order to screen for and detect BDD among their patients.

**Methods:**

This case-control study was conducted in the Faculty of Dentistry at Mashhad University of Medical Sciences, Mashhad, Iran. In total, 414 women were recruited between January 2019 and April 2020. After determining the ICON index, applicants filled out a demographic questionnaire, the Beck depression inventory (BDI II), Beck anxiety inventory (BAI), and Yale-Brown Obsessive-Compulsive Scale Modified for Body Dysmorphic Disorder (BDD-YBOCS).

**Results:**

In total, 31 (15%) cases in the orthodontics group and 21 (10.1%) subjects in the control group had a score of 20 or higher on the BDD-YBOCS (p=0.182). Moreover, there was no significant difference between groups in the mean BDD-YBOCS (p=0.184), BAI (p=0.163), and BDI-II (p=0.147). However, a statistically significant difference was found between the orthodontics patients and controls in the mean ICON index score (p<0.001). No correlation was found between the severity of ICON and BDD-YBOCS scores in all participants (p=0.804), cases (p=0.655), nor controls (p=0.403).

**Conclusions:**

Objective indices such as ICON were not able to screen for BDD. Furthermore, BDD has an increased prevalence in patients seeking orthodontic treatments. Orthodontists should look for BDD features in patients during the first visit by careful history taking and can benefit from utilizing the BDD-YBOCS survey as a screening tool in patients who are suspected of having BDD while referring the individuals who have higher scores to psychiatrists for further clinical evaluations.

**Disclosure of Interest:**

None Declared

